# BMI-1 is a potential therapeutic target in diffuse intrinsic pontine glioma

**DOI:** 10.18632/oncotarget.18002

**Published:** 2017-05-19

**Authors:** Shiva Senthil Kumar, Satarupa Sengupta, Kyungwoo Lee, Nanki Hura, Christine Fuller, Mariko DeWire, Charles B. Stevenson, Maryam Fouladi, Rachid Drissi

**Affiliations:** ^1^ Brain Tumor Center, Division of Oncology, Cincinnati Children's Hospital Medical Center, Cincinnati, OH, USA; ^2^ Division of Pathology and Laboratory Medicine, Cincinnati Children's Hospital Medical Center, Cincinnati, OH, USA; ^3^ Division of Pediatric Neurosurgery, Cincinnati Children's Hospital Medical Center, Cincinnati, OH, USA

**Keywords:** DIPG, BMI-1, cell proliferation, cancer stem cells, therapeutic target

## Abstract

Diffuse intrinsic pontine glioma (DIPG) is a poor-prognosis pediatric brain tumor. No effective curative therapy is currently available and no therapeutic advances have been made in several decades. BMI-1 is a member of the multimeric protein complex Polycomb repressor complex 1. It is highly expressed in a number of diseases and malignancies and has been implicated in self-renewal of normal and cancer cells, and in DNA damage signaling. The role of BMI-1 in DIPG is largely unknown. Here, we show that BMI-1 is highly expressed in tumor tissue samples of DIPG patients and in patient-derived cancer stem-like cells. BMI-1 downregulation leads to the inhibition of DIPG patient-derived neurosphere cell proliferation, cell cycle signaling, self-renewal, telomerase expression and activity, and suppresses DIPG cell migration. Moreover, targeted inhibition of BMI-1 sensitizes DIPG cells to radiomimetic drug-induced DNA damage. Together, our data validate BMI-1 as a potential therapeutic target to treat children with DIPG.

## INTRODUCTION

Brain tumors are the leading cause of cancer-related deaths in children [1]. DIPG represents 8-12% of pediatric central nervous system tumors [2, 3] and is the deadliest primary malignant brain tumors in children with a 3-year overall survival rate of 5-10% [4]. Hence, there is an urgent need to develop novel therapies that not only improve outcome, but mitigate long-term complications in children with DIPG.

Epigenetic modulation of histone proteins plays an important role in oncogenic transformation and regulation of gene expression in many cancer types. Recent studies have identified in DIPG a recurrent monoallelic somatic mutation in histone genes *H3F3A* and *HIST1H3B* encoding the replication-independent histone variant H3.3 and the canonical histone H3.1, respectively, resulting in a lysine-to-methionine change (H3.1/H3.3K27M) [5, 6]. The prevalence of mutations affecting these histones is reported in 80-93% of DIPG patients [7–10]. Lysine residues on histone H3 are often post-translationally modified to regulate chromatin structure and gene expression. Tri-methylation of H3K27 (H3K27me3) is catalyzed by the Polycomb-repressive complex 2 (PRC2). This repressive mark is recognized by the Polycomb complex, PRC1. PRC1 and PRC2 are large multimeric complexes involved in gene silencing through modifications of chromatin organization. The sequential histone modifications induced by PRC2 and PRC1 allow stable silencing of gene expression. The canonical human PRC1 is comprised of BMI-1 (B cell-specific Moloney murine leukemia virus integration site 1), RING1A/B, PCGF, CBX, and HPH. BMI-1 stimulates PRC1 E3 ligase activity by interacting and stabilizing the catalytic subunit RING1B. BMI-1 plays a major role in PRC1-dependent mono-ubiquitination of histone H2A at lysine 119 (H2A-K119Ub). BMI-1-associated E3 ubiquitin ligase activity represses multiple gene loci, including *INK4A/ARF* locus encoding for two tumor suppressors p16^INK4A^ and p14^ARF^ [11]. BMI-1 has been implicated in a number of biological functions including development, cell cycle, DNA damage response, senescence, stem cell proliferation and self-renewal and cancer [12]. Several studies have shown that BMI-1 is highly expressed in various cancer types and plays an oncogenic role by maintaining cancer cell stemness and self-renewal, promoting carcinogenesis, invasion and metastasis (reviewed in reference 12). Here we show that BMI-1 is highly expressed in DIPG and its downregulation leads to the inhibition of DIPG patient-derived stem-like cell proliferation, cell cycle signaling, self-renewal, telomerase expression and activity, and to the suppression of DIPG cell migration. Moreover, inhibition of BMI-1 expression sensitized DIPG cells to radiomimetic drug-induced DNA damage. Our data provide strong support for BMI-1 as a therapeutic target to treat patients with DIPG.

## RESULTS

### Increased BMI-1 expression in DIPG patient tissue and in patient-derived cell lines

**Figure 1:**
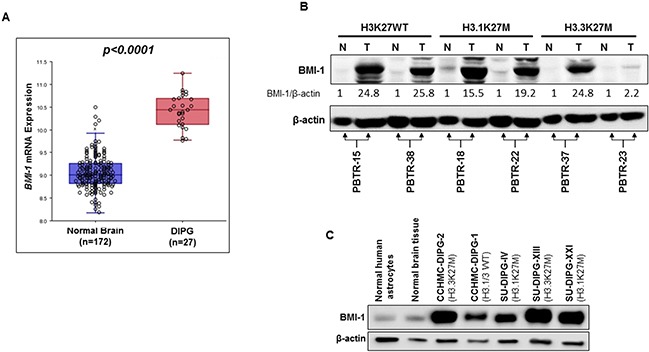
BMI-1 is highly expressed in DIPG tumors and patient-derived primary cell lines regardless of their H3K27M mutation status **(A)** *In silico* analysis of *BMI-1* mRNA expression in normal brain- and DIPG tissue. Each circle represents a tissue sample. *P* value is indicated. **(B)** Immunoblot analysis of BMI-1 expression in DIPG tumor (T), and matched normal (N) tissue from six DIPG patients. H3K27 mutation status is indicated (WT, wild type). Band intensities were quantified, normalized to β-actin, and are represented as values relative to respective matched normal. **(C)** Immunoblot analysis of BMI-1 expression in primary DIPG patient-derived neurosphere cell lines. The H3K27 mutation status is indicated. β-actin served as loading control.

We first performed *in silico* analysis of *BMI-1* mRNA expression in DIPG and in normal brain using the web based genomic analysis software R2 (http://r2.amc.nl) and publically available DIPG and normal brain gene expression datasets [13, 14]. We used the Megasampler module, which uses datasets from same chipsets (*u133p2*) and normalization methods (*MAS5.0*) allowing a fair comparison of gene expression. *BMI-1* mRNA expression was significantly higher (*p*-value<0.0001) in DIPG compared to normal brain tissue samples (Figure 1A). We then evaluated BMI-1 protein levels in six post-autopsy DIPG tumors and their matched normal brain tissue from different locations in the brain (Table S1). BMI-1 protein levels were markedly increased in all tumor tissues compared to their corresponding normal brain tissues (Figure 1B). Based on *H3F3A* and *HIST1H3B* K27 mutation status, the majority of DIPG tumors are distributed into three subtypes: H3.1K27M, H3.3K27M and wild-type (WT) H3.1/H3.3. Regardless of the H3 subtype, all DIPGs analyzed showed similar increased levels of BMI-1 protein except for one DIPG sample (PBTR-23). Similarly, BMI-1 protein level was also increased in DIPG tissue (PBTR-43) from a patient who did not undergo any treatment (Supplementary Figure 1), suggesting that the increased BMI-1 levels seen in DIPG is unlikely due to chemotherapy or radiotherapy. BMI-1 was further shown to be highly expressed in DIPG patient-derived primary neurosphere cell lines irrespective of *H3F3A* and *HIST1H3B* K27 mutation status (Figure 1C). These results suggest that increased BMI-1 protein levels might play an oncogenic role in DIPG.

### BMI-1 downregulation inhibits DIPG cell growth and neurosphere formation

DIPG patient-derived neurospheres showed high levels of BMI-1 protein, thereby providing an *in vitro* system to investigate the role of BMI-1 in DIPG and to test its validity as a therapeutic target.

**Figure 2:**
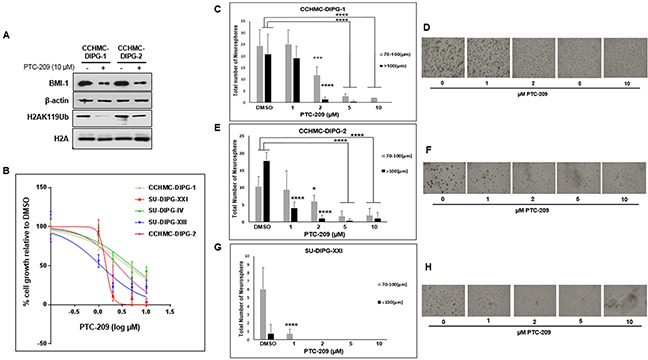
PTC-209 reduces BMI-1 levels, PRC1 activity, and inhibits cell growth of DIPG neurospheres **(A)** Immunoblot analysis of BMI-1 and H2AK119Ub levels following 72 h treatment with 10 μM PTC-209. β-actin and total H2A served as loading controls. **(B)** Cell proliferation of DIPG cell lines treated for 72 hours with various concentrations of PTC-209 (DMSO, 1, 2, 5, 10 μM), measured using WST-1 assay and represented as percent (%) cell growth relative to DMSO treatment (control). **(C–H)** Representative images and quantification of neurosphere size (μm) and number of DIPG neurospheres treated with the indicated concentrations of PTC-209. CCHMC-DIPG-1 (C-D), CCHMC-DIPG-2 (E-F), and SU-DIPG-XXI (G-H). Error bars represent the standard deviation obtained from triplicates. Each experiment was performed at least twice. *, *P* < 0.05; **, *P* < 0.01; ***, *P* < 0.001; ****, *P* < 0.0001.

PTC-209 is an investigational compound and is the first identified small molecule post-transcriptional inhibitor of BMI-1 [15]. Treatment with PTC-209 was shown to be specific to BMI-1, downregulating the protein levels in cancer cells and had no to limited effect on cell growth and viability in normal cells, indicating that PTC-209 activity is not due to cytotoxicity [15]. Treatment of DIPG neurospheres with PTC-209 led to a significant reduction of BMI-1 protein levels (Figure 2A). BMI-1 is a cofactor of RING1B, a specific monoubiquitination E3 ligase that ubiquitinates H2A at lysine 119, producing the chromatin repressive mark H2A-K119Ub [16]. As expected, the reduction in BMI-1 protein levels was associated with a global decrease of H2A-K119Ub mark with no effect on total H2A (Figure 2A). PTC-209 treatment inhibited the growth of a panel of DIPG neurosphere cell lines regardless of H3.1/H3.3 mutation status. The inhibition was dose- dependent with IC_50_ ranging from 1.8 to 4.5 μM (Figure 2B and Supplementary Table 2). Moreover, PTC-209 treatment not only severely limited the number of DIPG spheres but also reduced the sphere size (Figure 2C–2H). Together, these data indicate that the reduction of BMI-1 protein levels using a small molecule inhibits PRC1 activity and limits the ability of DIPG neurospheres to proliferate and self-renew.

### BMI-1 downregulation affects RB pathway and causes cell cycle arrest and cell death in DIPG cells

**Figure 3:**
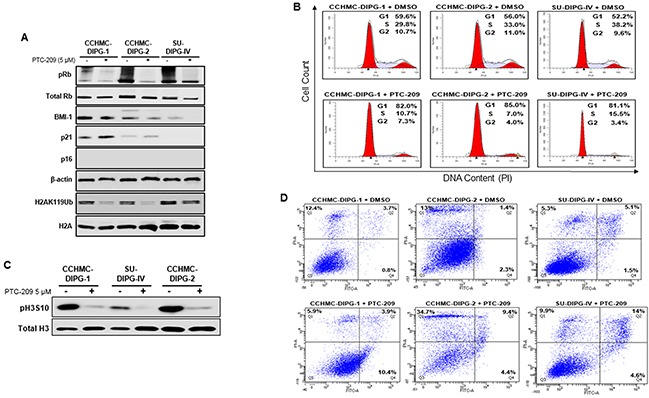
BMI-1 downregulation affects RB pathway, induces G_1_/S cell-cycle arrest and apoptosis in DIPG cells **(A)** Immunoblot analysis of pRB, total Rb, BMI-1, p21, p16^ink4A^ and H2AK119Ub extracted from DIPG cell lines treated with DMSO or PTC-209. β-actin and total H2A served as loading controls. **(B)** Cell cycle analysis of PTC-209 treated DIPG cells. DMSO treatment represents the control. Analysis was performed by gating on live cells only. Percentage of cells in G_1_, S and G_2_/M is indicated. **(C)** Immunoblot analysis of pH3S10, a marker of mitosis. Total H3 served as loading control. **(D)** Flow-cytometry analyses of PTC-209 or DMSO treated DIPG cells stained with annexin-V and propidium iodide (PI). The percentage of apoptosis (lower far right quadrant) and overall cell death (upper far right quadrant) is indicated. In all experiments, cells were either treated with DMSO or 5 μM PTC-209 for 3 days.

BMI-1-associated E3 ubiquitin ligase activity represses *INK4A/ARF* locus encoding for two tumor suppressors p16^INK4A^ and p14^ARF^ (in humans), thereby controlling self-renewal and cell cycle [17, 18]. The tumor suppressor p16^INK4A^ inhibits the CDK4/6 kinase activity resulting in hypophosphorylation and the suppression of RB activity leading to cell cycle arrest. The treatment of DIPG neurospheres with PTC-209 induced suppression of RB phosphorylation in all DIPG subtypes, and increased p21^WAF1/CIP1^ expression in CCHMC-DIPG-1 (H3.1/H3.3 WT) and CCHMC-DIPG-2 (H3.3K27M), (Figure 3A). Surprisingly, we did not observe an increase in p16^INK4A^ expression in any of the DIPG cells analyzed (Figure 3A and Supplementary Figure 2A-2B). BMI-1 is a transcriptional repressor shown to directly regulate *p21^WAF1/CIP1^* expression by binding to its promoter [19]. p21^WAF1/CIP1^ has been proposed as an alternate tumor suppressor that inhibits cell growth and the RB pathway by blocking CDK4/CDK6 activity [19] and RB phosphorylation through inhibiting cyclin E and CDK4 in neural progenitor cells [20]. Interestingly, PTC-209 treatment was able to reduce RB phosphorylation levels in SU-DIPG-IV without induction of p21^WAF1/CIP^. This could be explained by the fact that SU-DIPG-IV harbors a gain in MDM4 [21] which is involved in the 26S-proteasomal degradation of p21^WAF1/CIP^ in the G_1_ and early S phases [22]. Therefore, it is possible that p21^WAF1/CIP^ may have been induced by PTC-209 treatment but degraded due to the gain in MDM4 in SU-DIPG-IV cells. Confirming these molecular changes, cells treated with PTC-209 showed a significant accumulation in G_0_/G_1_ and subsequent decrease in S phase cell population (Figure 3B), indicating G_1_/S arrest. This arrest was further confirmed by a drastic decrease in the levels of the M phase marker pH3S10 (Figure 3C).

Next, we checked the effect of BMI-1 downregulation on DIPG cell viability. Treatment of DIPG neurospheres with PTC-209 for 72 hours induced 4-10% apoptosis in all DIPG cell types and an overall cell death of 14-19% (Figure 3D, quadrants Q2 and Q4). These results suggest that the cell growth inhibition was mainly due to G_1_/S cell cycle arrest.

**Figure 4:**
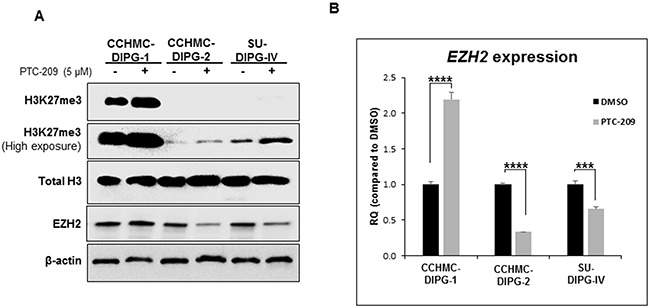
BMI-1 downregulation by PTC-209 leads to an increase in H3K27me3 levels **(A)** Immunoblot analysis of H3K27me3 and EZH2 from DIPG cell lines treated for 3 days with 5 μM PTC-209. β-actin and total H3 served as loading controls. **(B)** *EZH2* expression in DIPG cell lines treated for 3 days with 5 μM PTC-209 by qPCR. Error bars represent the standard deviation obtained from triplicates for each condition. Each experiment was performed at least twice. *, *P* < 0.05; **, *P* < 0.01; ***, *P* < 0.001; ****, *P* < 0.0001.

Tri-methylation of H3K27 (H3K27me3) is catalyzed by H3K27-specific histone methyltransferase, enhancer of zeste homologue 2 (EZH2), a subunit of PRC2. This transcriptionally repressive epigenetic mark is enriched at silent gene promoters in mammalian cells [23, 24] and plays an important role in regulating expression of developmentally regulated genes [25–27]. Unexpectedly, the treatment of DIPG neurospheres with PTC-209 induced an increase in the levels of H3K27me3 mark in all DIPG neurospheres regardless of the H3.1/H3.3K27 mutation status (Figure 4A). Although we do not fully understand the reason behind this increase, it seems to be independent of EZH2 protein and transcript levels (Figure 4A and 4B). These results suggest that BMI-1 downregulation-induced growth inhibition is due to the activation of cell cycle checkpoint via RB inactivation and *p21^WAF1/CIP1^* induction. To our knowledge, this is the first study showing that the RB pathway is a potential functional target in DIPG. Furthermore, BMI-1 downregulation is associated with an increase in the level of the repressive mark H3K27me3.

### BMI-1 downregulation is associated with diminished clonogenicity and stemness markers expression of DIPG neurospheres

**Figure 5:**
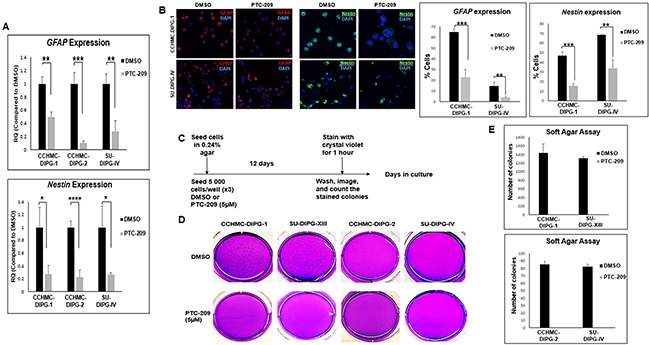
BMI-1 downregulation by PTC-209 affects stemness properties of DIPG **(A)** *GFAP* and *Nestin* expression in DIPG cell lines treated for 3 days with 5 μM PTC-209 by qPCR. Error bars represent the standard deviation from two independent experiments performed in triplicates. **(B)** Representative immunofluorescence images and quantificationsof GFAP (red) and Nestin (green) expression in CCHMC-DIPG-1 and SU-DIPG-IV cells treated for 3 days with 5 μM PTC-209, DAPI (blue). **(C–E)** Schematic of the soft-agar assay. Representative images and quantifications from soft agar assay comparing the colony forming ability of DIPG cells treated with for 12 days with 5 μM PTC-209. *, *P* < 0.05; **, *P* < 0.01; ***, *P* < 0.001; ****, *P* < 0.0001.

**Figure 6:**
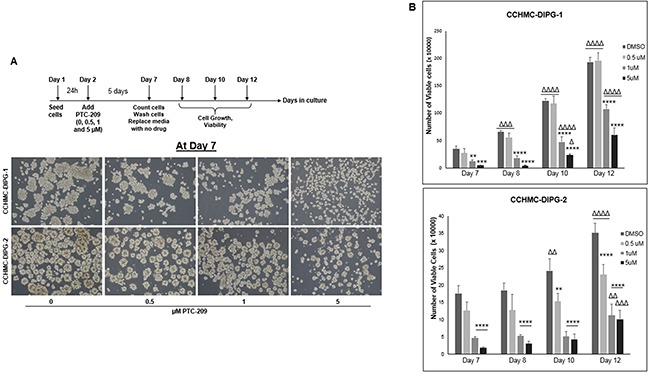
BMI-1 downregulation by PTC-209 irreversibly impairs the self-renewal capacity of DIPG cells **(A)** Schematic of the drug removal experiment and representative images of cells at Day 7 (5 days of continuous treatment). **(B)** Corresponding plots showing the quantifications of viable cell numbers after removal of PTC-209, at days 8, 10, and 12. Error bars represent the standard deviation from triplicates. *, *P* < 0.05; **, *P* < 0.01; ***, *P* < 0.001; ****, *P* < 0.0001. ^Δ^, *P* < 0.05; ^ΔΔ^, *P* < 0.01; ^ΔΔΔ^, *P* < 0.001; ^ΔΔΔΔ^, *P* < 0.0001.* denotes comparison between doses on the same day, ^Δ^ denotes comparison between days with the same dose.

BMI-1 was shown to maintain normal and cancer stem cells self-renewal phenotype [12]. In glioblastoma, BMI-1 plays an important role in cell proliferation and self-renewal of neural stem cells [12]. Therefore, we sought to evaluate the effect of BMI-1 protein level reduction on the stemness properties of DIPG neurospheres. We confirmed that under serum-free culture conditions, the DIPG neurospheres expressed neural stem cell markers such as nestin, CD133, and olig2, and were capable of self-renewal and differentiation in the presence of serum (Figure 5, and data not shown). Treatment with PTC-209 resulted in a significant decrease in the expression of both RNA and protein levels of GFAP and Nestin in all DIPG cell types tested regardless of H3.1/H3.3K27 mutation status (Figure 5A and 5B). Moreover, using the soft-agar colony forming assay, the downregulation of BMI-1 completely abolished the colony-forming ability of all DIPG cell types tested (Figure 5C–5E). Together, these data indicate that BMI-1 downregulation affects the proliferation and the self-renewal ability of DIPG cells, suggesting an impairment of DIPG cells’ stemness and tumorigenic potential. Moreover, to evaluate the reversibility of the PTC-209-mediated impairment of DIPG stemness, we treated DIPG cells for 5 days with PTC-209, and then replated the viable cells in drug-free medium and monitored cell growth for an additional 5 days. As expected, PTC-209 treatment decreased the viable cell number. This effect was sustained after drug removal as evidenced by the persistent decrease in viable cells initiated by the prior treatment with PTC-209, suggesting that the effect of BMI-1 inhibition on DIPG stem cells proliferation is sustained in a fraction of cells (Figure 6A and 6B). The remaining viable cells continued proliferating with no apparent difference in the growth rate between treated and untreated cells after PTC-209 removal (Figure 6B).

### PTC-209 treatment suppresses hTERT expression and telomerase activity

**Figure 7:**
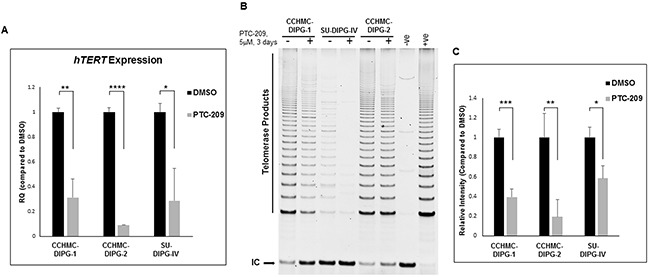
PTC-209 treatment decreases *hTERT* expression and telomerase activity in DIPG cells **(A)** *hTERT* expression in DIPG cells treated for 3 days with 5 μM PTC-209 by qPCR. **(B)** Telomerase activity in DIPG cells treated for 3 days with 5 μM PTC-209 measured by the TRAP assay. **(C)** Quantification of the TRAP assay represented as the mean ± S.D from independent experiments. (IC) PCR internal control; (−ve), TRAP negative control; (+ve), TRAP positive control. The intensity of telomerase products (6-bp ladder) and the 36-bp internal control (IC) bands were quantified using Image Studio (LI-COR biosciences). Relative telomerase activity was calculated as the intensity ratio of the TRAP ladder (telomerase products) to that of the PCR internal control. *, *P* < 0.05; **, *P* < 0.01; ***, *P* < 0.001; ****, *P* < 0.0001.

Cancer development and progression are closely associated with telomere length maintenance, which in most cases, results from the activation of telomerase, thought to be a critical step in cellular immortalization and carcinogenesis. We have previously shown that *hTERT* is upregulated in the majority of DIPG specimens tested [29]. BMI-1 was shown to prevent replicative senescence and to immortalize human mammary epithelial cells through the activation of telomerase [18]. Moreover, the elevated levels of BMI-1 expression correlate with an increase of telomerase activity in ovarian cancer tissues [30]. Telomerase activation is mediated by the expression of the rate-limiting determinant of the enzymatic activity, the catalytic subunit hTERT. We evaluated the effect of PTC-209-mediated decrease of BMI-1 protein level on *hTERT* expression and telomerase activity. PTC-209 treatment significantly decreased *hTERT* expression (Figure 7A) and telomerase activity (Figure 7B and 7C) in all DIPG cell lines tested regardless of H3.1/H3.3K27 mutation status. These results indicate that increased BMI-1 levels sustains *hTERT* expression and its inhibition may limit the proliferative capacity of DIPG cells through telomerase repression.

### PTC-209 targets EMT in DIPG cells

**Figure 8:**
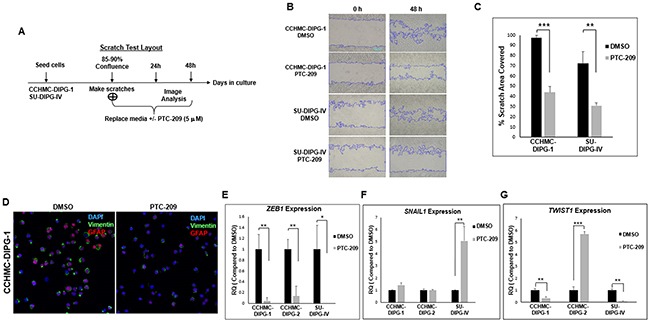
PTC-209 treatment inhibits the invasive properties of DIPG and downregulates the EMT factors ZEB1 and Vimentin **(A)** Experimental design of the scratch assay. **(B)** Representative images of the scratch assay at 0 and 48 hrs-time points post-treatment. **(C)** Quantifications of the scratch assay showing the percentage area covered in 48 hrs with after DMSO or PTC-209 treatment. **(D)** Representative immunofluorescence images GFAP (red) and Vimentin (green) expression in CCHMC-DIPG-1 and SU-DIPG-IV cells after treatment with DMSO 5 μM PTC-209 for 3 days, DAPI (blue). **(E–G)** *ZEB1, SNAIL1* and *TWIST1* expression in cells treated with DMSO or 5 μM PTC-209 for 3 days by qPCR. Error bars represent the standard deviation from three independent experiments performed in triplicates. *, *P* < 0.05; **, *P* < 0.01; ***, *P* < 0.001; ****, *P* < 0.0001.

We have previously shown the propensity of DIPG for aggressive local and distant metastatic spread [31]. Cell invasion is associated with cell migration and plays a key role in metastasis. Epithelial-mesenchymal transition (EMT) is a hallmark of cancer invasion and metastasis. EMT process is regulated by master regulators, including the transcription factors ZEB, TWIST and SNAIL. The function of these factors is regulated at the transcriptional, translational and post-translational levels. The involvement of these factors in EMT depends on the cell or tissue type and the signaling pathways that initiated EMT. They often control the expression of each other and functionally cooperate at target genes [32, 33]. BMI-1 was shown to be essential in the regulation of *SNAIL* expression in breast cancer and *TWIST1* expression in head and neck cancer [12]. We assessed the effect of BMI-1 inhibition on DIPG cells migration capacity using an *in vitro* scratch assay. Both CCHMC-DIPG-1 (H3.1/H3.3 WT) and SU-DIPG-IV (H3.1K27M) cells treated with PTC-209 displayed a significant decrease in migration into the scratch area (Figure 8A–8C). Vimentin expression is a marker of EMT and cancer metastasis [34]. Stable silencing of BMI-1 in invasive mesenchymal endometrial cancer cells was shown to downregulate vimentin, and significantly reduced cell invasion *in vitro* [35]. Treatment with PTC-209 led to a noticeable decrease in vimentin levels in DIPG cells (Figure 8D). Since migration and invasiveness are hallmarks of EMT, we investigated the possible mechanisms of EMT inhibition by PTC-209-induced BMI-1 inhibition. We evaluated the expression of transcription factors involved in EMT, TWIST, SNAIL and ZEB1. Treatment with PTC-209 led to a strong decrease in *ZEB1* expression in all DIPG cells tested regardless of H3.1/H3.3K27 mutation status (Figure 8E). PTC-209-induced BMI-1 inhibition had no striking effect on *SNAIL1* expression in CCHMC-DIPG-1 (H3.1/H3.3 WT) and CCHMC-DIPG-2 (H3K27M) cells, but induced a sharp increase in *SNAIL1* expression in SU-DIPG-IV cells (H3.1K27M, Figure 8F). Regarding *TWIST1*, BMI-1 inhibition induced a decreased expression in CCHMC-DIPG-1 and SU-DIPG-IV cells, but produced a robust increase in expression in CCHMC-DIPG-2 cells (Figure 8G). The significance of these findings is currently under investigation. Together, these results suggest that PTC-209-induced BMI-1 downregulation is effective in inhibiting the migration capacity of DIPG cells, possibly through the repression of the EMT master regulator *ZEB1*.

### PTC-209 sensitizes DIPG to Bleocin

**Figure 9:**
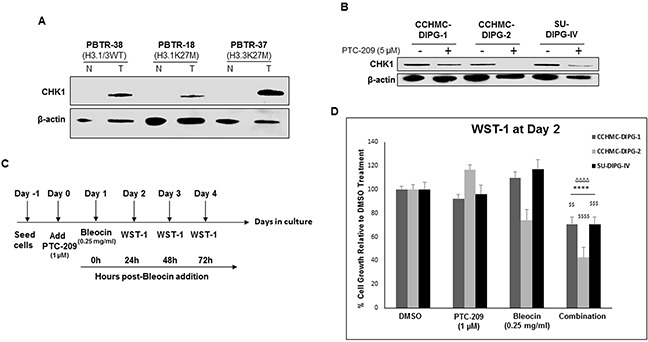
PTC-209 enhances radiosensitivity of DIPG cells to DNA damage **(A)** Immunoblot of CHK1 levels in DIPG patient tumor (T) and matched normal (N) tissue. **(B)** Immunoblot of CHK1 levels in DIPG cell lines treated for 3 days with 5 μM PTC-209. **(C)** Experimental design of the PTC-209 and bleocin combination treatment. **(D)** Cell growth measured by WST-1 assay, and represented as percent (%) of cell growth at 24 hrs compared to 0 h after addition of Bleocin. Error bars represent standard deviation from independent experiments each run in triplicates. *, *P* < 0.05; **, *P* < 0.01; ***, *P* < 0.001; ****, *P* < 0.0001. ^Δ^, *P* < 0.05; ^ΔΔ^, *P* < 0.01; ^ΔΔΔ^, *P* < 0.001; ^ΔΔΔΔ^, *P* < 0.0001. ^$^, *P* < 0.05; ^$$^, *P* < 0.01; ^$$$^, *P* < 0.001; ^$$$$^, *P* < 0.0001. * denotes comparison with DMSO, ^Δ^ denotes comparison with PTC-209 and ^$^ denotes comparison with Bleocin.

Cancer stem cells are thought to be responsible for cancer recurrence and therapy resistance. BMI-1 was shown to confer radioresistance to normal and cancerous neural stem cells through recruitment of the DNA double-strand break response machinery, and its downregulation sensitized cancer cells to ionizing radiation [36]. Recently, BMI-1 was shown to upregulate the multidrug resistance protein 1 (MDR1) upon cisplatin treatment of cells from different types of cancer [37]. Moreover, BMI-1 silencing enhanced cisplatin-therapy response in ovarian cancer [38]. ZEB1 was identified as a substrate of ATM phosphorylation, promoting radioresistance through stabilization of ZEB1 and CHK1 [39]. Furthermore, ZEB1 levels correlate with CHK1 protein levels and poor clinical outcome in human breast cancer [39]. In our DIPG patient tumors, we observed an increased CHK1 protein levels compared to their matched normal tissue (Figure 9A). As shown above, *ZEB1* expression was repressed upon PTC-209-induced BMI-1 downregulation (Figure 8E). This repression correlated with a significant decrease in CHK1 protein levels (Figure 9B). Next we checked whether CHK1 decrease sensitizes DIPG neurospheres to radiomimetic drug Bleocin. Indeed, pretreatment of DIPG neurospheres with PTC-209 sensitized all DIPG cells tested to a sublethal dose of Bleocin as early as 24 hours post-treatment (Figure 9D). Together, these results provide a proof of concept that PTC-209-induced BMI-1 downregulation holds the potential to sensitize DIPG cells to radiotherapy at least, in part by impeding the DNA damage response mediated by CHK1 downregulation.

## DISCUSSION

Our results showed that BMI-1 is highly expressed in the majority of DIPG specimens tested compared to the matched normal tissue. Importantly, it is highly expressed in all DIPG subtypes regardless of H3.1/H3.3K27 mutation status. Similarly, BMI-1 is highly expressed in all subtypes of patient-derived DIPG neurospheres. This finding indicates that *BMI-1* is a signature gene for DIPG and further supports the association of elevated BMI-1 protein levels with therapy failure and poor prognosis, a hallmark of DIPG. Cancer stem-like cells have been proposed to represent sub-populations of cells within a tumor that self-renew to promote tumor growth and progression. In the present study, we evaluated the role and the therapeutic value of BMI-1 in patient-derived DIPG stem-like cells. PTC-209 is a small molecule that targets BMI-1 protein production by tampering with its post-transcriptional regulation [40]. PTC-209 was shown to be specific to BMI-1 with limited to no effects on normal cells [12, 15]. Treatment of DIPG neurospheres with PTC-209 reduces BMI-1 protein levels and inhibited cell growth and neurosphere formation in a dose-dependent manner regardless of H3.1/H3.3K27 mutation status suggesting that (1) BMI-1 has an oncogenic role in DIPGs, required for tumor growth, and (2) BMI-1 is a valid therapeutic target for DIPG. Since the *INK4A* and *ARF* locus is rarely mutated in DIPG, we were expecting *INK4A* induction and pRB hypophosphorylation. Surprisingly, *p16^INK4A^* was not induced, instead pRB hypophosphorylation was associated with *p21^WAF1/CIP1^* induction in two DIPG cell lines. These data suggest that p16^INK4A^-RB pathway is not functional in our DIPG cells, and that BMI-1 promotes DIPG cell proliferation by repressing *p21^WAF1/CIP1^* expression, indicating that p21-RB axis is a potentially actionable therapeutic target in DIPG. We do not rule out the possibility that *p21^WAF1/CIP1^* repression may not be the only mechanism by which pRB phosphorylation is induced. As a result of PTC-209-induced pRB hypophosphorylation, G_1_/S arrest was induced with 14-19% of cells undergoing cell death, indicating that PTC-209-induced-cell growth inhibition was essentially caused by G_1_/S arrest. The EZH2-independent increase in the levels of H3K27me3 mark upon treatment with PTC-209 is intriguing. It could be the result of K27 demethylase JMJD3 inhibition. Interestingly, drug-mediated inhibition of JMJD3 was shown to increase cellular H3K27 methylation in DIPG cells and to be therapeutic for DIPG [28].

BMI-1 protein level reduction led to the repression of stem cell markers indicating that BMI-1 regulates DIPG stemness. Furthermore, BMI-1 downregulation affects the anchorage-independent clonogenicity and the self-renewal ability of DIPG cells, suggesting an impairment of DIPG cells’ stemness and tumorigenic potential. The drug withdrawal experiments indicate that the impairment of the self-renewal ability of DIPG cells seems to be persistent. Moreover, PTC-209-induced BMI-1 downregulation may limit DIPG proliferation capacity by inhibiting telomerase via *hTERT* repression.

PTC-209 treatment decreased the migration capacity of DIPG cells. TWIST1 and SNAIL1 were shown to cooperate in the induction of *ZEB1* expression [41]. However, we did not see a consistent effect of BMI-1 down-regulation on *SNAIL1* and *TWIST1* expression. These results suggest that BMI-1 induces EMT in DIPG through *ZEB1* up-regulation and possibly through SNAIL1 or TWIST1 depending on the DIPG subtype.

The role of BMI-1 in DNA damage response has been reported in several studies [12, 42, 43]. Here we show that CHK1 protein levels are high in DIPG tumor tissue and the decrease of BMI-1 levels is associated with a decrease in CHK1 protein levels. This finding is consistent with the potential role of the axis BMI-1-ZEB1-CHK1 in DIPG radioresistance and therefore therapy failure.

In conclusion, we provided data to support the oncogenic role of BMI-1 in DIPG and its potential as a therapeutic target for the treatment of this devastating disease. We shed light on various cellular processes involving BMI-1. It is critically important to determine the exact role of BMI-1 in DIPG development and progression.

Our studies set the stage for the preclinical and clinical testing of BMI-1 inhibitors such as PTC-596 in DIPG. PTC-596 is more potent [44] and is currently in Phase I clinical trial in adults with advanced solid tumors.

## MATERIALS AND METHODS

### Tissue samples, cell lines and drugs

The ATCC CRL-2091 normal primary human foreskin fibroblast strain (HFF) and the HeLa human cervical carcinoma cell line were obtained from the American Type Culture Collection.

Tissue samples were obtained as described previously [31]. Briefly, patients with DIPG were informed and consented to the IRB-approved Pediatric Brain Tumor Repository (PBTR) autopsy protocol (Study: 2013-1245) at Cincinnati Children's Hospital Medical Center. Diagnosis of DIPG was based on clinical symptoms and imaging characteristics on pre-treatment magnetic resonance imaging (MRI). DIPG tumor tissue and matched normal samples (n=7) were evaluated in the present study. CCHMC-DIPG-1 and CCHMC-DIPG-2 are primary neurosphere cell lines, derived from DIPG patient tissue specimens as previously described [21]. SU-DIPG-IV, SU-DIPG-XIII and SU-DIPG-XVII, primary neurosphere cell lines, were kind gifts from Dr. Michelle Monje (Stanford University). All neurosphere cell lines were grown in stem cell culture media and propagated as non-adherent cultures as previously described [21] except for SU-DIPG-IV, which was grown as adherent culture. H3.3 and H3.1 mutation status for tissue and neurosphere cell lines was determined as previously described [31]. Normal human astrocytes were immortalized with SV40 large T antigen. PTC-209 (Selleck Chemicals) was reconstituted in DMSO. Bleocin (Sigma) was reconstituted in sterile water.

### R2: Genomics analysis and visualization platform

*In silico* comparison of BMI-1 mRNA levels were performed using the online genomics analysis software, R2 (http://r2.amc.nl) developed by the department of Oncogenomics in the Academic Medical Center (AMC) Amsterdam, Netherlands. Expression of BMI-1 mRNA was compared between normal brain [13] (n=172, GEO ID:GSE11882) and DIPG autopsy tumor tissue [14] (n=27, GEO ID:GSE26576). One-way Anova was used to analyze the data.

### Western blotting

DIPG neurospheres were dissociated using TrypLE express (GIBCO, USA) and lysed using RIPA buffer (100 mM NaCl, 10 mM Tris at pH 7.5, 0.1% SDS, 0.5% Deoxycholate, 1% NP40, 1X proteinase inhibitor cocktail from Roche and 50 mM NaF) on ice for 30 min. Following centrifugation at 13 000 x g for 20 min, (i) the supernatant was used as whole cell lysate and quantified using Bio-Rad protein assay (Bio-Rad, USA). 15 μg of protein was separated using SDS-PAGE; (ii) the pellet was treated with 1X laemmli buffer supplemented with β-mercaptoethanol and heated at 95°C for 5 min to extract histones. The sample was centrifuged briefly and the supernatant was used to blot histones. An equivalent volume corresponding to 40,000 cells was used to load equal amounts of histones. Following SDS-PAGE, proteins were transferred to a nitrocellulose membrane, blocked with 5% milk in TBST for 1 hr at room temperature, and incubated with respective primary antibodies. Antibodies used were against p16^INK4a^/CDKN2A (ab108349) and RB (ab24) purchased from Abcam; H3K27me3 (#9733), Chk1 (#2360), EZH2 (#5246), p21^WAF1/CIP1^ (#2947), Phospho-Rb (Ser807/811) (#9308), β-Actin (#3700), Bmi1 (#6964), Ubiquityl-Histone H2A (Lys119) (#8240), Phospho-Histone H3 (Ser10) (#3377), Histone H2A (#3636) and Histone H3 (#3638) purchase from Cell signaling. The blots were incubated with their respective horseradish peroxidase conjugated anti-mouse (#31430) or anti-rabbit (#31460) secondary antibody (Thermo Scientific) for 1 hr at room temperature. Bands were visualized using ECL with Azurec500 imaging system (Azure Biosystems). Band intensities were quantified using Image Studio (Ver. 4, LI-COR Biosciences).

### WST-1 cell proliferation assay

Neurospheres were dissociated to single cells, and 5 000 cells were seeded in triplicates, with DMSO or desired concentrations of PTC-209. After 72 hrs, WST-1 reagent (Takara Bio, USA) was added to the wells (1:10 final dilution) incubated for 1 hr at 37°C. Absorbance was measured at 450 nm wavelength with 650 nm as the reference.

### Neurosphere size and number measurement

Neurospheres were dissociated and seeded as single cells in triplicates, with DMSO or PTC-209 for 72 hrs. Images of neurospheres were taken using an inverted light microscope (Leica, Germany). ImageJ software (imagej.nih.gov) was used to measure the number and size of neurospheres by manually drawing a ruler across the widest diameter of a given neurosphere.

### Real time PCR

Total RNA was extracted from cells using RNAzol (MRC, Inc, USA) following manufacturer's instructions. 1 μg of RNA was converted to cDNA using qScript cDNA SuperMix (Quanta Biosciences, USA). Taqman probes for *GAPDH* (Hs_00266705_g1), *GFAP* (Hs_00909233_m1)*, NESTIN* (Hs_04187831_g1)*, OLIG2* (Hs_00377820_m1)*, hTERT* (Hs_00972650_m1), *p16^INKA^* (Hs_02902543_mH) and *EZH2* (Hs_05448333_m1) were purchased from Applied Biosystems (ABI, USA), and qPCR was performed using PerfeCTa® qPCR FastMix® II (Quanta Biosciences, USA). Primers for *TWIST1* (F: 5′- GGACAAGCTGAGCAAGATTCAGA-3′ and R: 5′-GTGAGCCACATAGCTGCAG-3′)*, SNAIL1* (F: 5′-CCTCAAGATGCACATCCGAAG-3′ and R: 5′-ACATGGCCTTGTAGCAGCCA-3′), and *ZEB1* (F: 5′-GGCAGAGAATGAGGGAGAAG-3′ and R: 5′-CTTCAGACACTTGCTCACTACTC-3′) were purchased from Integrated DNA Technologies (IDT, USA) and qPCR was performed using Power SYBR™ Green Master Mix (ThermoFisher Scientific, USA). PCR condition followed was: 95°C for 10 min; 95°C for 10 s, 59°C for 20 s, 72°C for 30 s (45 cycles)[45]. *GAPDH* was used for normalization. Relative expression (represented as RQ) was obtained using the Comparative C_T_ (ΔΔC_T_) method.

### Scratch assay

Performed as previously described [46]. Briefly, CCHMC-DIPG-1 and SU-DIPG-4 cells were used for this assay. Stem cell media complemented with 1% FBS was used to grow CCHMC-DIPG-1 as adherent cells. Cells were allowed to grow up to 80-90% confluency. Two perpendicular scratches were made with a P1000 pipette tip, washed twice with PBS and replaced with media containing either DMSO or PTC-209. Images were taken every 24 hrs. Scratch area covered was measured using ImageJ software (imagej.nih.gov).

### Soft agar assay

The assay was performed in six well plate with each condition (DMSO or PTC-209) in triplicates. Neurospheres were dissociated, and 5 000 single cells were seeded in culture medium (with or without PTC-209) containing 0.24% Noble agar (Difco, USA) on top of a solidified 0.4% agar layer. After 12 days, the colonies (spheres in agar) were stained with 0.05% crystal violet solution (Sigma, USA) at room temperature for 1 hr. After washing the wells with water for 3-4 times to remove the background staining, images were captured for each well, and crystal violet -positive (dark blue) colonies were counted for quantification.

### Immunofluorescence

Neurospheres treated with DMSO or PTC-209 were dissociated into single cells, and were cyto-spun to attach on glass-slides. Cells were then fixed with 4% paraformaldehyde (PFA), washed with PBS, blocked for non-specific binding using blocking solution (PBS supplemented with 5% donkey serum and 0.3% Triton X-100) and incubated overnight with the indicated primary antibodies [anti-GFAP (Z0334) and anti-Vimentin (M7020), DAKO; anti-Nestin (MAB5326, Millipore)]. After PBS wash the next day, the cells were incubated with secondary antibodies (Alexa-Fluor-594 conjugated Donkey anti-Rabbit (#711-585-152) and Alexa-Fluor-488 conjugated Donkey anti-Mouse (#715-545-150); Jackson ImmunoResearch) for 1 hour, and washed with PBS. Finally, the slides were embedded with Mounting Media with DAPI (Vector Laboratories, USA). Images were captured at 60X oil objective in confocal microscope (Nikon, USA).

### Cell cycle analysis

Following treatment with DMSO or PTC-209, neurospheres were dissociated into single cells, washed with cold PBS, and were fixed with ice-cold 70% ethanol for at least 30 minutes in ice. Cells were again washed with cold PBS and were treated with 100 μg/ml Ribonuclease-A (Sigma, USA) for 20 min at room temperature followed by the addition of propidium iodide (PI) (25 μg/ml; Sigma, USA) in the dark. The stained cells were subsequently analyzed by BD FACS Canto II flow cytometer. Data were analyzed by BD FACSDiva v6.1.3 software (BD Biosciences, USA) and Modifit (ModFit LT; BD, USA).

### Apoptosis assay

The assay was conducted using the Annexin V Apoptosis Detection kit (#88-8007, Affymetrix) according to manufacturer's instructions. Briefly, 1 × 10^6^ cells in 100 μl of 1X binding buffer were stained with fluorochrome-conjugated Annexin V (5 μl) and propidium iodide (5 μl) in 200 μl of 1X binding buffer for 15 min and were then analyzed by flow cytometry Canto 2 (BD FACSCanto™ II; BD Biosciences, Franklin Lakes, NJ, USA).

### Telomeric repeat amplification protocol (TRAP)

TRAPeze Telomerase detection kit (Millipore) was used to evaluate telomerase activity. TRAP was performed according to the manufacturer's protocol using 50 ng of proteins. The final PCR products were run on a 12.5% polyacrylamide gel, stained with SYBR safe DNA gel stain (Thermo Fisher), and were visualized using a Gel Imager (Typhoon FLA7000, GE). Band intensities were measured using Image Studio (Ver. 4, LI-COR Biosciences).

### Statistical analysis

Multiple *t* tests and ANOVA analysis were used to analyze data. For multiple comparisons, ANOVA followed by a *post-hoc* Tukey or Dunnet's test was performed whichever applicable. GraphPad Prism 6.0 (GraphPad Software, Inc., USA) was used for the above mentioned statistical analyses.

## SUPPLEMENTARY MATERIALS FIGURES AND TABLES


