# Supplementary material for: BMI-1 is a potential therapeutic target in diffuse intrinsic pontine glioma
Source: Oncotarget. 2017 May 19;8(38):62962–75. doi: 10.18632/oncotarget.18002 (PMC5609895; doi:10.18632/oncotarget.18002)
Supplement: Supplementary file 1 [file oncotarget-08-62962-s001.pdf]

BMI-1 is a potential therapeutic target in diffuse intrinsic pontine glioma

SUPPLEMENTARY MATERIALS

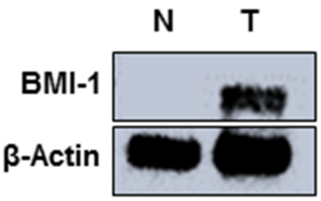

Supplementry Figure 1: BMI-1 is highly expressed in post-autopsy DIPG tumor tissue from patient with no prior radiation or chemotherapy. Immunoblot analysis of BMI-1 expression in DIPG tumor PBTR-43. (T), tumor, (N) matched normal.

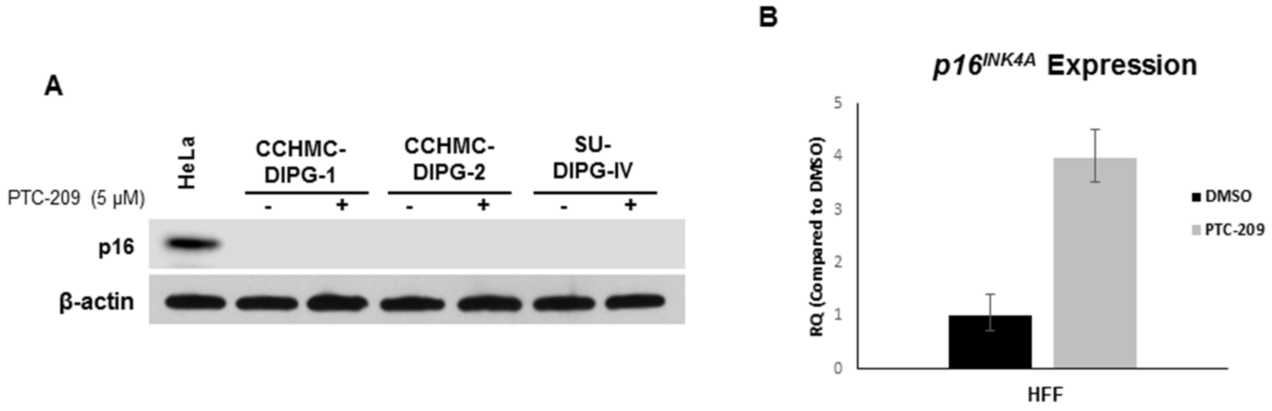

Supplementry Figure 2: *p16^INK4A* is not induced upon BMI-1 downregulation in DIPG cells. (A) Immunoblot analysis of p16^{INK4A} expression in HeLa cells (positive control) and in DIPG cells treated with 5 µM of PTC-209 for 72 hrs. **(B)** Treatment of normal primary human foreskin fibroblasts (HFF) with 5 µM PTC-209 for 72 hrs induced *p16^{INK4A}* expression assessed by qPCR. Error bars represent the standard deviation from two independent experiments performed in triplicates.

Supplementry Table 1: Anatomic locations of the matched normal tissue from DIPG patients (PBTR)

Patients	Matched normal tissue location
PBTR-15	Frontal cortex
PBTR-18	Frontal cortex
PBTR-22	Right frontal lobe
PBTR-23	Right cerebellum
PBTR-37	Left frontal lobe
PBTR-38	Right frontal lobe
PBTR-43	Right frontal lobe

Supplementary Table 2: IC_{50} (µM) values of PTC-209 in DIPG cell lines

Cell Lines	IC_{50} (µM)
CCHMC-DIPG-1	3.996
SU-DIPG-XXI	1.927
SU-DIPG-IV	4.505
SU-DIPG-XIII	1.820
CCHMC-DIPG-2	2.551
